# Translating the Results of a Selective Decontamination of the Digestive Tract Trial into Efficacious Real-Life Interventions

**DOI:** 10.37825/2239-9747.1038

**Published:** 2022-12-27

**Authors:** Ornella Piazza, Giovanni Boccia, Carolina Ciacci, Francesco De Caro, Amelia Filippelli, Gianluigi Franci, Pasquale Pagliano

**Affiliations:** Department of Medicine, Surgery, Dentistry “Scuola Medica Salernitana”, University of Salerno, Via Allende, Baronissi, SA, Italy

## Dear Editor

We read with great interest the recently published SuDDICU trial [1] and the metanalysis regarding the Association Between Selective Decontamination of the Digestive Tract and In-Hospital Mortality in Intensive Care Unit Patients Receiving Mechanical Ventilation by Hammond et al. [2]. Both papers highlight the importance of adding an intravenous agent as a component of the intervention and demonstrate that Selective Decontamination of the Digestive Tract (SDD) is a safe treatment, that does not cause an increase presence of antibiotic-resistant organisms, and C. difficile infections raise.

After reading the articles, the Sepsis Group of the Department of Medicine, Surgery, Dentistry of the University of Salerno (an ICU physician, a pharmacologist, a gastroenterologist, a microbiologist, an infectious disease, and two Hygiene and Public Health specialists) met to discuss how to implement SDD in our clinical practice. We wanted to share our thoughts on translating the results of the SuDDICU trial into efficacious real-life interventions. However, further relevant information regarding the study should be considered prior to applying the results of the study to our patients. The discussion led us to recognize that we missed but needed to include some information. The first is how to relate the SuDDICU trial low levels of antimicrobial resistance (23.1% vs. 34.6% in the study group and control group, respectively) with our academic ICU higher rate of prevalence of antimicrobial resistance. Moreover, we noted the different percentages of surgical patients, which in our ICU will be greater than the 27,6% of the SuDDICU trial series, and our different rates of cardiac surgery patients, which represent only 3% of the whole population of the SuDDICU. Differences may be relevant, as a previous retrospective study showed that cardiosurgical patients preoperatively receiving tobramycin and polymyxin orally did not show beneficial effects on the clinical outcomes [3]. According to us, a pharmacokinetic study on the chosen antimicrobials should be integrated in the protocol. Third, we considered the feasibility of giving patients scheduled for postoperative ICU admission after elective surgery the oral paste before intubation. Concerns were raised about the palatability of the oral paste and reduced access to the upper gastrointestinal tract for gastric suspension when the nasogastric tube is removed. Finally, we wondered if a microbiome study would point out possible short- and long-term modifications of the intestinal flora [4].

Even if the possibility of the emergence of anti-microbial-resistant organisms is very unclear in our setting and undoubtedly scary, the chance that the use of SDD in patients receiving mechanical ventilation in the ICU may reduce the incidence of ventilator-associated pneumonia and new positive blood cultures is still very appealing. Our analysis is ongoing, and we look for hints and suggestions to understand how to apply this relevant research to our everyday practice.
